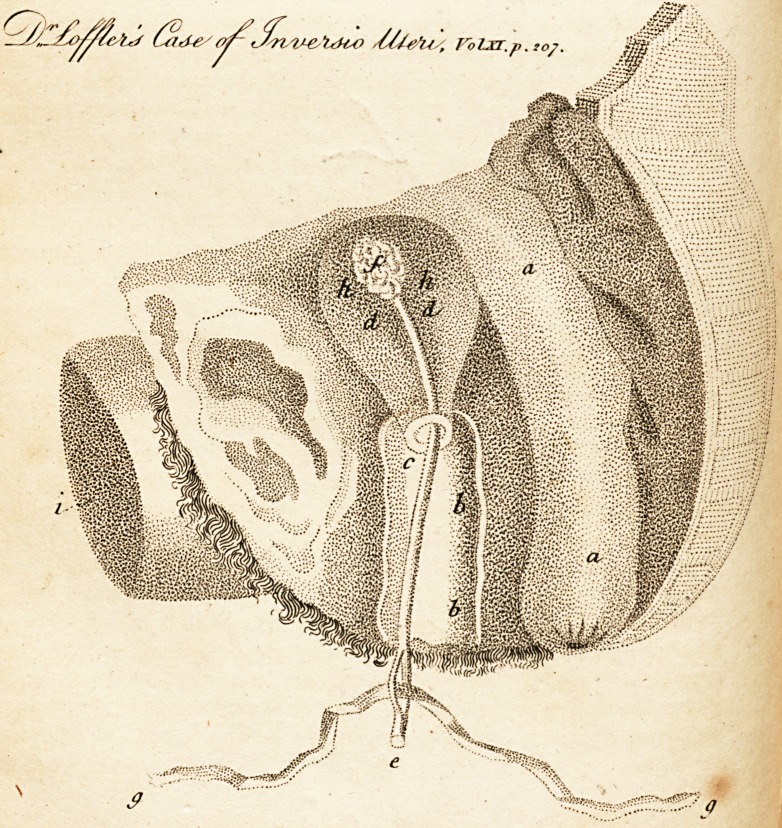# Case of an Inversio Uteri

**Published:** 1804-03-01

**Authors:** 

**Affiliations:** Petersburg


Dr. Loffler's Case of Inversio Uteri, Vol XI. p. 207.

### Case of an Inversio Uteri. *By* Dr. Loffler, *of Petersburg.*

[ With an Engraving. ]

A Woman of a bloated and full habit, mother of several children, received in her last lying-in, by the rough and improper extraction of the placenta, an inversio uteri; but as it was soon replaced by a skilful surgeon, and as the woman was ordered to keep for some time a situation on the back, the uterus perfectly contracted, and remained in its natural situation; shortly after she left the bed, and went to her usual work. In the course of two years she became again pregnant, and during the whole time she enjoyed the best health till the proper time of delivery, which went on rather quickly; and the midwife, instead of protracting it, imprudently accelerated it as much as possible. What had occurred in the preceding delivery took place in this; and the fundus uteri being inverted, descended to the orificium uteri. It was however fortunate that the midwife discerned this accident, and I was accordingly called to her assistance. On examination I found the uterus to have passed through the orifice, and I felt in the vagina a soft and bloody tumour, *(completa partialis inversio.)* The placenta had passed away, and it was a happy circumstance for the patient, that the hæmorrhage was only moderate. As the orifice of the uterus had a little contracted itself, the uterus became slightly incarcerated. I immediately attempted to replace the uterus in the horizontal situation in which I found the woman, but should this not succeed, I intended to lay her forward on the knees and elbows, in which position I certainly hoped to perform the reposition. I attained my purpose however not without some difficulty and pains, by the following method. Having introduced my right hand into the vagina, I spread my fingers round the tumour formed by the uterus, so that the fundus rested in the hollow hand; and having previously compressed it gently, and drawn it a little forward, I endeavoured to push it back by degrees and with gentleness, in which, after some repeated efforts, I at last succeeded. The fundus uteri having thus receded through the orifice of the uterus, I pursued it with my hand, which I kept in the uterus, waiting for the uniform contraction of this organ. But after I had continued in this position above half an hour, without perceiving any contraction, I was obliged to withdraw my hand, when the fundus immediately descended, though it was prevented by the patient's posture on the back with the pelvis elevated, and by introducing a sponge which had been provided with a string into the orifice to pass through it. This circumstance made me think of a perfect atonia of the uterus, which could easily have been avoided if the woman had been delivered in a horizontal posture, in which her labours would not have been so violent, and if the waters had been sooner discharged discharged, and the head of the child suffered to pass through by degrees. After the patient had reposed a little, I applied cold injections of vinegar and water, which mixture was also often thrown by means of a syringe on the belly; but as this remedy would not produce the least contraction, I had recourse to stronger stimulants, injections of brandy, of salt water, and of water of ammonia, ordering at the same time the belly to be rubbed with vinegar, and giving excitant remedies internally. All my endeavours however were ineffectual, and after having continued these applications above twelve hours the uterus remained in the same state as before.

I now desisted from the application of external and internal excitantia, and also from the employment of stronger ones, being fearful of bringing on an inflammation, which I knew from experience to be always dangerous in an atonic or paralytic organ; and adopted a mechanical expedient for removing the inversion; and though I could have prevented a total inversion and prolapsus out of the vagina by means of a pessarium, I could not acquiesce in having recourse to it, before I had tried another expedient which presented itself to my mind, and which I flattered myself would be capable of effecting a perfect cure, by keeping the fundus uteri at a distance from the orifice and by elevating it, so as to render the uterus fit for contraction with the assistance of proper remedies, and thus to make a radical cure. For this purpose I procured a tube, made of horn, long enough to reach up to the fundus uteri; it was bent according to the axis of the pelvis, and had two holes on the upper extremity, by means of which a sponge could be fastened; at *a*, the lower extremity, another hole was made, through which a string was drawn, which could be fastened to a bandage round the belly, whereby the instrument was kept in its proper situation. With this instrument I gently pushed the fundus uteri upwards, and supported it in the proper situation. This operation being performed without much difficulty, and attended with little pain, the patient felt no kind of inconvenience, and the lochiæ passed off with ease. By making this support of the uterus hollow, and by providing it at the upper end with several holes, I had the advantage of being able to make injections into the uterus without difficulty, which was performed twice a day, with a decoction of flores arnica, in order to excite the paralytic powers of that organ. When this had been continued several days, during which the patient remained quietly in bed, she felt some pains, which gradually increasing, became exactly like those of a woman in labour, whereby the uterus contracted, till the instrument was entirely protruded out of the uterus, and I could take it easily away. On examination, I found the uterus perfectly contracted and the orifice shut. I ordered injections into the vagina to be continued with a decoction of willow (cort. salic. fragil.) The patient gradually recovered her health and strength, and was completely cured of the complaint.

EXPLANATION OF THE PLATE.

*a. a.* The intestinum rectum.
*b. b.* The vagina.
*c.* The orifice of the uterus.
*d. d.* The uterus.
*e.* The lower extremity of the instrument with which the uterus is supported.
*f.* The upper extremity where the sponge is fastened.
*g. g.* The string by which the instrument is fastened.
*h. h.* Openings in the hollow supporter, through which the injected liquor passes.
*i.* Part of the thigh.

## Figures and Tables

**Figure f1:**